# Input Estimation for Extended-Release Formulations Exemplified with Exenatide

**DOI:** 10.3389/fbioe.2017.00024

**Published:** 2017-04-19

**Authors:** Magnus Trägårdh, Michael J. Chappell, Johan E. Palm, Neil D. Evans, David L. I. Janzén, Peter Gennemark

**Affiliations:** ^1^School of Engineering, University of Warwick, Coventry, UK; ^2^Cardiovascular and Metabolic Diseases, Innovative Medicines and Early Development Biotech Unit, AstraZeneca, Mölndal, Sweden; ^3^Global Product Development, Pharmaceutical Technology and Development, AstraZeneca, Mölndal, Sweden; ^4^Department of Systems and Data Analysis, Fraunhofer-Chalmers Centre, Gothenburg, Sweden

**Keywords:** input estimation, deconvolution, Markov chain Monte Carlo, exenatide, extended release

## Abstract

Estimating the *in vivo* absorption profile of a drug is essential when developing extended-release medications. Such estimates can be obtained by measuring plasma concentrations over time and inferring the absorption from a model of the drug’s pharmacokinetics. Of particular interest is to predict the bioavailability—the fraction of the drug that is absorbed and enters the systemic circulation. This paper presents a framework for addressing this class of estimation problems and gives advice on the choice of method. In parametric methods, a model is constructed for the absorption process, which can be difficult when the absorption has a complicated profile. Here, we place emphasis on non-parametric methods that avoid making strong assumptions about the absorption. A modern estimation method that can address very general input-estimation problems has previously been presented. In this method, the absorption profile is modeled as a stochastic process, which is estimated using Markov chain Monte Carlo techniques. The applicability of this method for extended-release formulation development is evaluated by analyzing a dataset of Bydureon, an injectable extended-release suspension formulation of exenatide, a GLP-1 receptor agonist for treating diabetes. This drug is known to have non-linear pharmacokinetics. Its plasma concentration profile exhibits multiple peaks, something that can make parametric modeling challenging, but poses no major difficulties for non-parametric methods. The method is also validated on synthetic data, exploring the effects of sampling and noise on the accuracy of the estimates.

## 1 Introduction

Extended-Release (ER) drug formulations are commonly used to improve the properties of drugs. They can allow for less frequent dosing schedules, improving compliance and quality for the patient. They can also improve safety by lowering the peak plasma concentration and enable the development and use of drugs whose pharmacokinetic (PK) properties would otherwise be unacceptable. For ER medications, the formulation design is specifically intended to provide a targeted release or input rate that optimizes the compound PK. ER medications are typically administered orally (tablets and capsules) or injected as intramuscular/subcutaneous depot formulations.

Most types of oral ER technologies today are based on polymeric systems (Yang and Pierstorff, 2012; Arafat, 2015). The oral formulations can be categorized into matrix, reservoir (or membrane controlled), and osmotic systems (Ratnaparkhi and Gupta Jyoti, 2013; Khalane et al., 2016). The drug release mechanisms involve drug diffusion, system swelling, or erosion and dissolution, or osmotic pressure-induced release (Siepmann and Göpferich, 2001; Arifin et al., 2006). Parenteral depot injections are used to achieve extended drug release over a week or longer. They include formulation types such as oil-based solutions, drug suspensions, polymer-based microspheres and polymer-based or lipid liquid crystal *in situ* formings (Rhee et al., 2010; Gulati and Gupta, 2011; Schwendeman et al., 2014). Biodegradable microsphere systems (e.g., made of PLGA copolymer) have proved to be a successful approach to deliver macromolecular drugs (Mitragotri et al., 2014).

In any ER-formulation development process, it is fundamental to determine the *in vivo* drug release/absorption profile of each candidate formulation. This is done routinely in drug discovery and development. Measuring the absorption profile *in vivo* is generally difficult and expensive. Typically, the data that are available are plasma concentration profiles following extravascular administration. If a model of the PK is available, it is possible to infer the absorption profile from plasma concentration data. The total amount of drug absorbed, and therefore the bioavailability, can be computed by integrating the absorption profile. Standard methods exist for the case where the PK is linear (Verotta, 1996). However, methods that are applicable to the non-linear case are not widely available.

When predicted *in vivo* input profiles are available, it may be possible to validate or invalidate the translatability of the *in vitro* system. Given data for several candidate formulations, an *in vitro in vivo correlation* (ivivc) can be established, relating the *in vitro* drug dissolution or release to the *in vivo* drug absorption or release (Lu et al., 2011; Cardot and Davit, 2012). Ideally, one can then predict the *in vivo* performance based on the *in vitro* release profile and optimize the formulation by *in vitro* testing at low cost. In addition, knowledge of the absorption profile in an animal model can help in predicting, and hence optimizing, the human PK profile. To achieve this, a human intravenous PK model is required, either from real data or predicted from cellular or animal data. The absorption profile obtained from animal data is fed to the human model, resulting in human PK trajectories. This type of human predictions is always desired in drug discovery to assess feasibility. Naturally, prediction reliability increases with the amount and quality of data.

One way to estimate the absorption profile is to build a parametric model of the drug release and absorption processes. For the drug release process, various models have been proposed, ranging from simple empirical models to detailed mechanistic models that account for various processes such as degradation and erosion (Siepmann and Peppas, 2001; Versypt et al., 2013). However, if the release profile is complicated, it may be difficult to create a model that is able to capture the observed plasma concentration (Shen and Burgess, 2015). One example is long acting biodegradable particles for subcutaneous injection. The model may also need to be tailored to the particular type of drug and formulation used. For sparse data, such models may also have practical identifiability issues. An alternative is to use *non-parametric* methods. In these methods, the release/absorption profile is allowed to take any functional form as long as it matches the data and does not exhibit any unrealistic behavior, such as taking negative values. Predictions from such non-parametric methods are often sufficient for compound/formulation selection in drug discovery.

This paper considers such non-parametric methods for estimating the release/absorption profile and bioavailability of extended-release formulations and gives advice on the choice of methods, given the data and system knowledge that are available. The choice of method depends on the characteristics of the PK model:

- When the dynamics of the PK model are substantially faster than the release/absorption profile, it is reasonable to assume that the PK model is essentially in steady state over the timescales of interest. The plasma concentration at any timepoint is a function of the absorption rate only at that timepoint, regardless of previous history. For linear PK models, the relationship between plasma concentration and absorption rate is linear.
- When the dynamics of the PK model are too slow to be ignored in relation to the absorption profile, the plasma concentration at any timepoint is a function of the complete absorption profile up to that point. If the PK model is linear and time-invariant, the relationship between the absorption rate *u*(*t*) and plasma concentration *C*(*t*) is given by
  (1) C(t)=I(t)∗u(t),
  where *I*(*t*) is the impulse response of the system and ∗ is the *convolution operator*. Estimating *u*(*t*) from *C*(*t*) is consequently referred to as *deconvolution* (Verotta, 1996). The impulse response can be derived from a model, if one is available, or may be determined empirically, e.g., from intravenous data.
- The most general case is when the dynamics of the PK model are non-linear. Here, the relationship between *u*(*t*) and *C*(*t*) cannot be expressed by a convolution operation. Estimating the absorption profile is still possible if a (non-linear) PK model is available. In this case, the dynamics are represented by a system of ordinary differential equations, which is integrated numerically as part of the estimation procedure. Since this operation is not related to convolution, we prefer the more general term *input estimation*.

**Figure 1:**
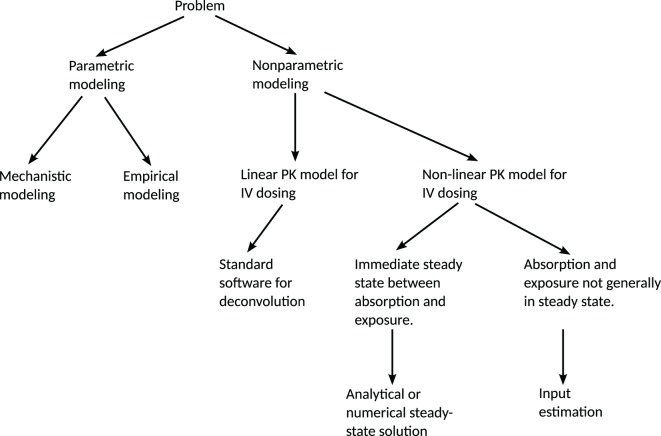
**Decision tree for the choice of method for predicting a release/absorption profile**. If a suitable model for the release/absorption profile exists or can be constructed, parametric methods are suitable. Otherwise, non-parametric methods can be used. The method presented in this paper addresses the most general non-parametric case, with non-linear PK and without any steady-state assumptions. If linearity or steady-state conditions can be assumed, simplifications can be made in order to reduce the computational requirements. IV = intravenous administration.

A decision tree summarizing these aspects is given in Figure 1.

In its most general form, *input estimation* is the technique of estimating the input to a dynamical system, given measurements of the system’s state. In the present case, the input is the release/absorption profile of the drug, *u*(*t*), the dynamical system is the PK model, and the measurements are of plasma concentrations *C*(*t*). A methodology for performing such analyses has been presented in Trägårdh et al. (2016). These methods do not make any assumptions about stationarity or linearity and are therefore applicable to the most general case presented above. Previously, the methods have been applied to estimating the absorption profile of an immediate-release (IR) formulation of the drug eflornithine as well as for estimating the energy intake in bodyweight models. The purpose of this paper is to evaluate the applicability of the method of Trägårdh et al. (2016) to ER-formulation problems and to investigate what adaptations, if any, are necessary in order to ensure good performance on this kind of problem. Additionally, the accuracy of the method is evaluated on simulated data for which the true input function is known. Estimation of ER release/absorption profiles differs from estimation of IR profiles in the following respects:

1. The timescales of ER absorption profiles can vary over large ranges, potentially much larger than the time constants of the PK model.
2. The absorption profile of ER formulations is typically considerably more complicated than the absorption profile of IR formulations.

Model dynamics that are fast compared to the timescales of interest can cause stiffness issues. In general, an estimation method that has previously been shown to perform well will not necessarily perform well when applied to a problem with substantially different data and model parameters. For this reason, it is essential to evaluate the methodology in Trägårdh et al. (2016) on a realistic ER estimation problem.

In this paper, these methods are applied to Bydureon (Buse et al., 2010, 2013), an extended-release microsphere formulation of the GLP-1 receptor agonist exenatide (Buse et al., 2004; DeFronzo et al., 2005). The Bydureon formulation consists of exenatide encapsulated within poly-(d,l-lactide-co-glycolide) (PLG) microspheres that are designed to release exenatide over an extended period of time which allows once-weekly patient-administered subcutaneous injections (European Medicines Agency, 2011). Typical *in vitro* release curves for Bydureon are given in Figure 3 in DeYoung et al. (2011). Such curves can be used, together with predicted input profiles from *in vivo* data, to establish an ivivc. In humans, Bydureon exhibits a multiphasic concentration–time profile over approximately 10 weeks consistent with the proposed mechanism of release from PLG microspheres. This is characterized by a limited initial rapid release of loosely bound surface exenatide (<1% released in the first few hours) followed by two additional phases corresponding to diffusion and erosion release with peak plasma concentrations at around week 2 and week 7 (DeYoung et al., 2011).

**Figure 2:**
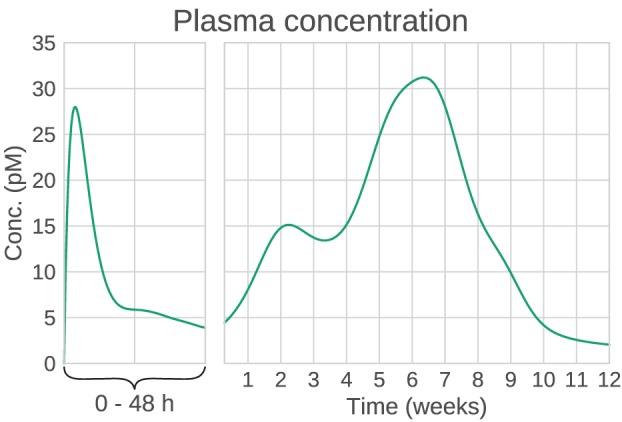
**Example plasma concentration profile of Bydureon, after a single dose of 10 mg**. This is a mean prediction from data obtained from the dose finding study by Fineman et al. (2011). Note that the clinical dose is 2 mg, but the characteristics of the PK profile are most clearly seen for the 10 mg dose. The profile shows multiple peaks, making the absorption rate non-trivial to model using conventional compartmental models.

The reason for choosing Bydureon as an example is that it is a drug that is already on the market, and data (Fineman et al., 2011; Li et al., 2015) as well as PK models (Gao and Jusko, 2012) are available in the literature. The complicated absorption profile of exenatide (Figure 2) cannot be easily captured by a simple parametric model. A compartmental model of the release and absorption processes has been proposed (Li et al., 2015), where the ER process is modeled by a cascade of transition compartments, and the initial amount of several compartments is non-zero. However, this model was designed to fit data from multiple-dosing experiments, where the multiple absorption peaks are not as noticeable.

The outline of the paper is as follows:

1. As a first step, the input-estimation method is validated. For this, it is necessary that the true input function is known. Therefore, simulated data are used. Additionally, real data tend to be sparse and noisy. Testing the method only on real data makes it difficult to determine whether any estimation error is due to problems with the method, or limitations on the data themselves. For this reason, estimation is first performed on ideal (densely sampled, noise-free) data. Only then is validation performed on simulated data with realistic sampling schedules and noise levels. The simulated data are generated by applying an Erlang distribution function as an input to the PK model, resulting in data similar to what is observed in actual experiments. The estimation method itself does not make any assumptions about the functional form of the absorption profile.
2. Once the method is validated, it is applied to real data from a dose-finding study.

## 2 Materials and Methods

### 2.1 Model

The PK model of Gao and Jusko (2012) was used for the system dynamics. The model includes non-linear target-mediated drug disposition (TMDD) and is given by

(2) $$\begin{array}{llll} \frac{\text{d}C\left(t\right)}{\text{d}t} & =\frac{u\left(t\right)}{V_{c}}-\left(k_{\mathrm{el}}+k_{\mathrm{pt}}\right)\cdot C\left(t\right)+k_{\mathrm{tp}}\cdot \frac{A_{T}\left(t\right)}{V_{c}}\; & & \;\\ & \;-k_{\mathrm{on}}\cdot \left(R_{\mathrm{tot}}-\mathrm{RC}\left(t\right)\right)\cdot C\left(t\right)+k_{\text{off}}\cdot \mathrm{RC}\left(t\right),\; \end{array}$$

(3) $$\frac{\text{d}A_{T}\left(t\right)}{\text{d}t}=k_{\mathrm{pt}}\cdot C\left(t\right)\cdot V_{c}-k_{\mathrm{tp}}\cdot A_{T}\left(t\right),$$

(4) $$\frac{\text{d}\mathrm{RC}\left(t\right)}{\text{d}t}=k_{\mathrm{on}}\cdot \left(R_{\mathrm{tot}}-\mathrm{RC}\left(t\right)\right)\cdot C\left(t\right)-\left(k_{\mathrm{off}}+k_{\mathrm{int}}\right)\cdot \mathrm{RC}\left(t\right),$$

where *C*(*t*) is the drug concentration in the central compartment, *A_T_*(*t*) is the drug amount in a peripheral compartment, *RC*(*t*) is the concentration of the drug–receptor complex, and *u*(*t*) is the unknown input. The parameter values reported in Gao and Jusko (2012) were used (Table 1). The model structure is similar to the PK model for eflornithine by Johansson et al. (2013), which was used for evaluating the estimation method on IR formulations in Trägårdh et al. (2016). The main difference between these model structures is that the model used here has an additional elimination mechanism in the form of drug-receptor complex internalization, represented by *k_int_* in equation (4). Additionally, the parameter values are substantially different.

**Table 1:** **Pharmacokinetic parameters, from Gao and Jusko (2012)**.

Parameter	Definition	Value	Unit
*k_el_*	Elimination rate constant	0.013	min^−1^
*k_pt_*	Intercompartmental rate constant	0.0685	min^−1^
*k_tp_*	Intercompartmental rate constant	0.0846	min^−1^
*V_c_*	Central volume of distribution	111	ml⋅kg^−1^
*k_on_*	Second-order binding constant	0.000411	pM^−1^⋅min^−1^
*k_off_*	First-order dissociation constant	0.566	min^−1^
*k_int_*	Internalization rate constant	0.00342	min^−1^
*R_tot_*	Total receptor concentration	1,240	pM

In addition to the model presented in Gao and Jusko (2012), similar models have been developed by Li et al. (2015) and Chen et al. (2013). These models differ from that of Gao and Jusko in that the total receptor concentration is described by a turnover model, instead of a fixed amount. Additionally, the Li et al. model was estimated using data from an ER formulation, using a linear 5-compartment model to represent the combined release and absorption process, while the Chen et al. model was estimated using data from an IR formulation and applying a Michaelis–Menten absorption function. As the input-estimation methods considered here provide non-parametric estimates of the release/absorption profiles, no parametric model was used in this paper for the release or absorption process. Instead, only the part of the PK model that describes the system dynamics following absorption was used, as detailed in equations (2)–(4).

To ensure the validity of the reported parameter values of these PK models, the structural identifiability was analyzed using the Exact Arithmetic Rank approach (Karlsson et al., 2012). The analysis showed that the PK models in Li et al. (2015) and Gao and Jusko (2012) are structurally identifiable. This result holds for intravenous (IV) administration, either bolus or continuous, as well as for subcutaneous (SC) administration. Since the model in Chen et al. (2013) shares the model structure of the PK after absorption with Li et al. (2015), it follows that this too is identifiable for IV administration. To summarize, any of these models could have been used in the subsequent analysis.

Once the PK parameters were shown to be identifiable, the next step was to ensure that the absorption profile can be estimated from plasma concentration data, given known PK parameters. An analysis of the identifiability of the input signal in the PK model in Gao and Jusko (2012) was performed using a Taylor series approach (Pohjanpalo, 1978). The analysis found that the input function is identifiable, given that the PK parameters are known. Details of the structural identifiability analysis of the PK models and the input function can be found in Section 1 in the Supplementary Material.

### 2.2 Estimation Method

The analysis in this paper uses the methods described in Trägårdh et al. (2016). In order to ensure that the methods were suitable for the ER estimation problem at hand, the following adaptations were performed:

1. This dataset posed a potential challenge for the input-estimation method: during the initial 48 h after the start of treatment, the plasma concentration was sampled relatively densely. Following this initial part, sampling was performed approximately once a week for 12 weeks. A very large number of basis functions may be required in order to capture the fast initial dynamics and at the same time cover the full 12-week period. This was solved by performing input estimation separately on the initial 48 h (short timescale) and on the full 12-week study (long timescale).
2. Two models for the likelihood were tested: one with a Gaussian and one with a Student’s *t*-distribution. The Student’s *t*-distribution with a small number of degrees of freedom is often suggested as an alternative to the Gaussian distribution, as it is less sensitive to outliers and therefore can result in a more robust inference (Gelman et al., 2014). This was found to be helpful for this dataset, as a Gaussian likelihood proved to be very sensitive to outliers in the data.

As described in Trägårdh et al. (2016), these input-estimation methods model the absorption profile as a stochastic process, which is equipped with a prior whose role is to discourage solutions that have unrealistically large oscillations (Verotta, 1996; De Nicolao et al., 1997). In any given estimation problem, a choice has to be made for

1. Choice of prior: for this analysis, a prior penalizing the *L*^2^ norm of the second derivative of the input function was chosen [equation (7) in Trägårdh et al. (2016)]. This choice enforces a relatively large degree of smoothness. To impose non-negativity constraints, the function was modeled in the log domain (Pillonetto et al., 2002).
2. Choice of functional representation: the input function was discretized into 20 basis functions based on the Karhunen–Loève expansion (Levy, 2008), as this was deemed to be sufficient to capture reasonable absorption profiles, while keeping the dimensionality of the estimation problem low.
3. Desired statistical quantities: for this analysis, it was desired to recover the full posterior distribution, in order to provide estimates of the uncertainty.
4. Choice of estimation algorithm: as a full posterior distribution was desired, estimation was performed using Markov chain Monte Carlo (MCMC) sampling (Metropolis et al., 1953; Hastings, 1970; Brooks et al., 2011).

Here, the *regularization parameter*, which determines the trade-off between the data fit and the smoothness conditions, was treated as a parameter to be estimated, being assigned a Gamma prior distribution with parameters α = β = 10^−3^. MCMC Samples were drawn by alternately updating the basis function coefficients using the Simplified Manifold Metropolis-adjusted Langevin algorithm (SMMALA) (Girolami and Calderhead, 2011), and updating the regularization parameter using Gibbs sampling (Geman and Geman, 1984). The Raftery–Lewis method (Raftery and Lewis, 1992) was used to assess the number of samples required, estimating the quantiles *q* = [0.025 0.25 0.5 0.75 0.975] with precision *r* = [0.02 0.05 0.06 0.05 0.02] and probability *s* = 0.95, as defined in Raftery and Lewis (1992).

It can be noted that on timescales of weeks, the system can be considered to be in a steady state. This means that the plasma concentration depends only on the current absorption rate, and the dynamical ODE model can be simplified to an algebraic model. This is done by setting all derivatives to zero, and solving for the measured quantity. This gives

(5) $$\begin{array}{llll} C(t) & =\frac{1}{2V_{c}k_{\mathrm{el}}k_{\mathrm{on}}}(-R_{\mathrm{tot}}V_{c}k_{\mathrm{int}}k_{\mathrm{on}}-V_{c}k_{\mathrm{el}}k_{\mathrm{int}}-V_{c}k_{\mathrm{el}}k_{\mathrm{off}}\; & & \;\\ & \;+u\left(t\right)k_{\mathrm{on}}+(R_{\mathrm{tot}}^{2}V_{c}^{2}k_{\mathrm{int}}^{2}k_{\mathrm{on}}^{2}+2R_{\mathrm{tot}}V_{c}^{2}k_{\mathrm{el}}k_{\mathrm{int}}^{2}k_{\mathrm{on}}\; & & \;\\ & \;+2R_{\mathrm{tot}}V_{c}^{2}k_{\mathrm{el}}k_{\mathrm{int}}k_{\mathrm{off}}k_{\mathrm{on}}-2R_{\mathrm{tot}}V_{c}u\left(t\right)k_{\mathrm{int}}k_{\mathrm{on}}^{2}\; & & \;\\ & \;+V_{c}^{2}k_{\mathrm{el}}^{2}k_{\mathrm{int}}^{2}+2V_{c}^{2}k_{\mathrm{el}}^{2}k_{\mathrm{int}}k_{\mathrm{off}}+V_{c}^{2}k_{\mathrm{el}}^{2}k_{\mathrm{off}}^{2}\; & & \;\\ & \;+2V_{c}u\left(t\right)k_{\mathrm{el}}k_{\mathrm{int}}k_{\mathrm{on}}+2V_{c}u\left(t\right)k_{\mathrm{el}}k_{\mathrm{off}}k_{\mathrm{on}}+u{\left(t\right)}^{2}k_{\mathrm{on}}^{2}{)}^{\frac{1}{2}}). \end{array}$$

**Figure 3:**
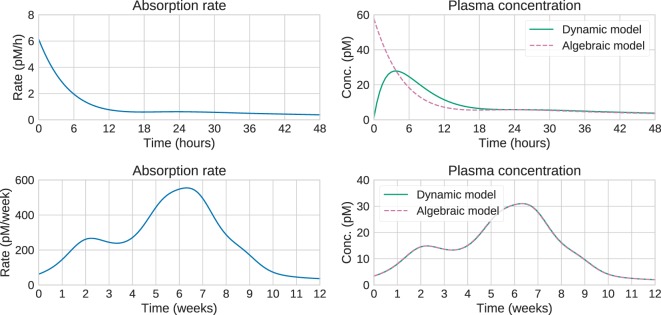
**Comparison of algebraic and dynamic models for short timescales (top) and long timescales (bottom)**. For a given release/absorption profile (left), the plasma concentration predicted by the algebraic and dynamic models is shown on the right. On short timescales, the predictions differ substantially. In this case, the steady-state approximation is not valid, and the “Input estimation” options in Figure 1 should be used. On longer timescales, the predictions are identical, suggesting that the computationally cheaper algebraic model can be used. This is the “Analytical or numerical steady-state solution” option in Figure 1.

Large computational savings can be achieved by utilizing this result instead of integrating the system of ODEs. This is especially valuable when using MCMC methods, which need to perform these computations a large number of times, potentially thousands. Figure 3 shows that for long timescales, the predictions from the dynamic and algebraic models are virtually identical, with relative differences of the order of 1%.

### 2.3 Simulated Data

A suitable synthetic input function was chosen based on the following criteria:

- It should result in data similar to those that were actually observed (see Section 2.4).
- It should have a simple functional form.
- It should ideally be the solution to a system of ODEs representing a compartmental model. The reason for this is that parametric PK models are usually compartmental models, and functions generated this way should therefore be able to capture realistic absorption behavior. This also makes it possible to give the function a model structure interpretation.

It can be noted that a compartmental model for the release/absorption process has been reported (Li et al., 2015). However, this model was not able to capture the peaks present from a single dose. Instead, it was found that datasets similar to the real data by Fineman et al. (Section 2.4) could be created by the following function:

- Each of the two peaks at longer timescales could be modeled as an Erlang distribution:
  (6) $$u_{i}\left(t\right)=a_{i}\frac{k_{tr_{i}}^{n_{i}}\cdot t^{n_{i}-1}\cdot e^{-k_{tr_{i}}t}}{\left(n_{i}-1\right)!},\;i=\left\{1,2\right\},$$
  where $k_{tr_{i}}$ is a rate constant that controls the rise and fall time of the peak, while *n_i_* largely controls the time delay.
- The initial absorption rate over short timescales was modeled as a bi-exponential function:
  (7) $$u_{3}\left(t\right)=r_{1}e^{-k_{1}t}+r_{2}e^{-k_{2}t}.$$
- The final input function *u*(*t*) was given by:
  (8) $$u\left(t\right)=u_{1}\left(t\right)+u_{2}\left(t\right)+u_{3}\left(t\right).$$

This function can be interpreted as the output of a compartmental model, as commonly used in PK modeling. This is explained in greater detail in Section 2 of the Supplementary Material. This methodology of evaluating a non-parametric method using parametric test functions is similar to that presented by Madden et al. (1996), the main difference being that the test function here was specifically designed to mimic the Bydureon profile, with initial fast dynamics followed by multiple peaks at longer timescales.

### 2.4 Real Data

The data for this analysis are from a study by Fineman et al. (2011). In the study, 54 subjects in 5 dose groups were given a single dose of exenatide. The plasma concentration was measured at 12 timepoints during the first 48 h, and subsequently once per week for a total duration of 12 weeks.

## 3 Results

### 3.1 Method Validation Using Simulated Data

To test the input-estimation method on simulated data, the test input function described in Section 2.3 was used, with the parameter values shown in Table 2. The parameters were selected to generate plasma concentrations similar to those observed in real data for the 10 mg dose.

**Table 2:** **Parameter values for the generated test data**.

Parameter	Value	Unit	Parameter	Value	Unit
*n*_1_	10	–	*r*_1_	0.12	pmol⋅min^−1^
*a*_1_	2,700	pmol	*r*_2_	0.02	pmol⋅min^−4^
$k_{tr_{1}}$	1.5 × 10^−4^	min^−1^	*k*_1_	7.64 × 10^−3^	min^−1^
*n*_2_	4	–	*k*_2_	4.76 × 10^−4^	min^−1^
*a*_2_	800	pmol			
$k_{tr_{2}}$	1.8 × 10^−4^	min^−1^			

Test data were generated by applying this input function to the dynamical system, and extracting the plasma concentration values at a set of timepoints. For each of the long and short timescales, two sampling schedules were used: one very dense, with 100 equally spaced points between *t* = 0 and the last timepoint (48 h for short timescales, 12 weeks for long), and one sparse using the same timepoints as in the real datasets. Additionally, two noise models were used: no noise and 10% proportional Student’s *t*-distributed noise with four degrees of freedom. The noise model was chosen to be equal to that assumed for the real datasets (see Section 3.2). This resulted in eight combinations of timescale, sampling schedule and noise levels. The number of MCMC samples was determined by the Raftery–Lewis diagnostics, which showed that all parameters for all datasets could be determined using 10,000 samples.

**Figure 4:**
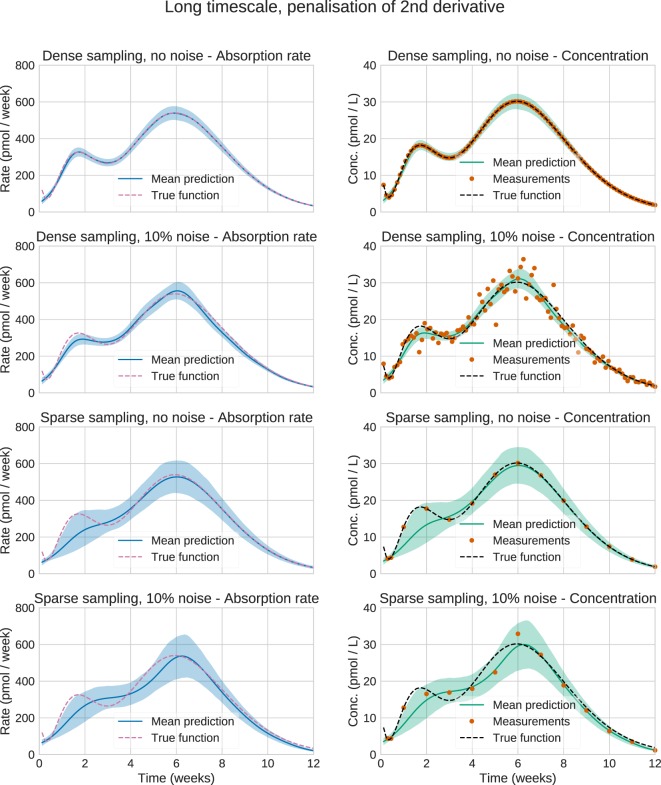
**Estimation results for the synthetic data**. Solid lines are mean predictions, while shaded areas are 95% credible intervals. It can be seen that the sizes of the credible intervals are affected strongly by the sparsity of the data, while noise has a considerably smaller effect. In all cases, the credible intervals mostly cover the true functions.

Figure 4 shows the plots for the long timescale response. From the figure, it can be seen that the method performs accurately on dense noise-free data. The exception is at the very first timepoint, which has a large contribution from the initial fast peak from *u*_3_(*t*). The performance was assessed by computing the root mean square error (RMSE) of the input function (Table 3). One RMSE value was computed for each trajectory sampled by the MCMC sampler, and the results were averaged. This way, the performance criterion accounts for the variance of the estimated input functions—an estimate where most of the posterior density is concentrated close to the true function will yield a lower mean RMSE than an estimate with a large variance.

**Table 3:** **RMSE (root mean square error) for the test datasets**.

Timescale	Measurement type	RMSE	Unit
Short timescale	Dense sampling, no noise	0.069	pmol/h
	Dense sampling, 10% noise	0.10	pmol/h
	Sparse sampling, no noise	0.14	pmol/h
	Sparse sampling, 10% noise	0.16	pmol/h
Long timescale	Dense sampling, no noise	14	pmol/week
	Dense sampling, 10% noise	23	pmol/week
	Sparse sampling, no noise	48	pmol/week
	Sparse sampling, 10% noise	50	pmol/week

### 3.2 Input Estimation on Real Data

**Figure 5:**
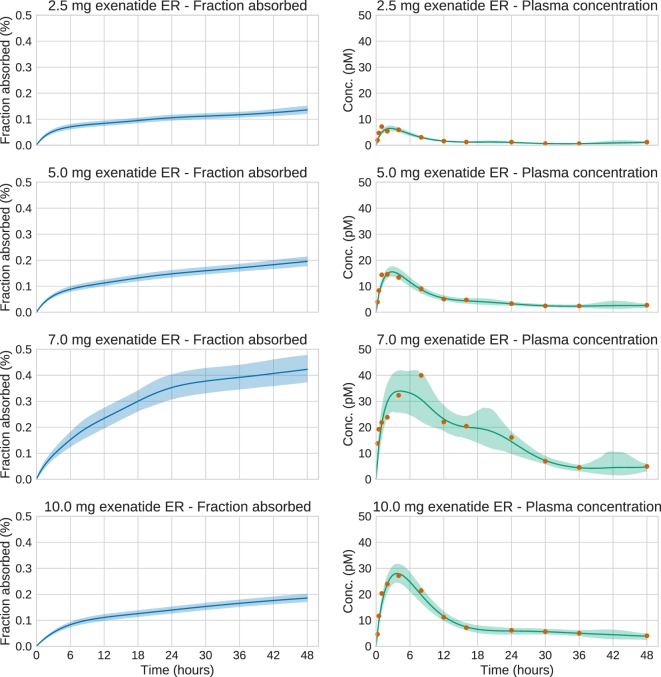
**Estimation results for the initial 48 h of the real exenatide data**. For most doses, the credible intervals are narrow. The main exception is the 7.0 mg dose. For this dose, the estimation method sets the regularization parameter to a low value in order to account for the large variations in the data. This increases the uncertainty of the estimate.

**Figure 6:**
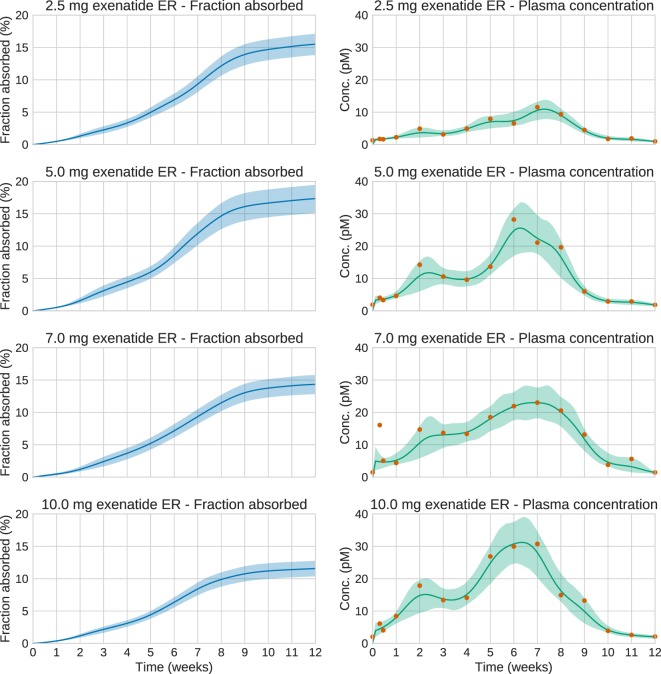
**Estimation results for the whole 12-week period of the real exenatide data**. Solid lines are mean predictions, while shaded areas are 95% credible intervals. At these timescales, the system is essentially at steady state. Note that the very large plasma concentration value in the first week for the 7.0 mg dose is treated as an outlier by the estimation method. This is a consequence of using an error model based on the Student’s *t*-distribution.

We now turn to the analysis of real Bydureon data from Fineman et al. (2011). From now on, the input will be shown as the fraction absorbed rather than the absorption rate, as this is the most common way to present such results. In contrast, when validating against test data, the absorption rate is more useful, since it shows features in the estimated absorption profile more clearly. Figure 5 shows the data and estimates for the initial 48 h. On this timescale, a dynamic model is necessary. Figure 6 shows the data and estimates over longer timescales. All plots are produced using a Student’s *t*-distribution with four degrees of freedom for the residual model. To assess the sensitivity to the number of degrees of freedom, inference was also performed using six degrees of freedom, which resulted in only marginal differences. The Raftery–Lewis diagnostics showed that 10,000 MCMC samples were enough for all datasets except for the 7 mg dose, which required 25,000 samples for short timescales and 40,000 for long timescales.

**Figure 7:**
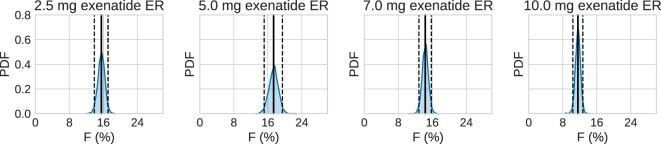
**Estimated bioavailability for the long-term release/absorption profiles**. The contribution from the initial peak is very small in comparison to the long-term release. The calculations assume 90 kg bodyweight, according to the data in Fineman et al. (2011). Solid lines are means, and dashed lines contain the 95% credible interval. Most noteworthy is the fact that the bioavailability drops for the highest doses.

Using the estimated profiles, the total absorbed amount of the drug, and hence the bioavailability *F*, was estimated. The amount absorbed during the first 48 h was determined to be insignificant compared to the total amount. Therefore, only the longer timescale was used. Figure 7 shows kernel density estimates for *F* for each dose group, where clearly *F* appears to be dose dependent. In all cases, the results are lower than the previously reported values of 22–25% (European Medicines Agency, 2011).

## 4 Discussion

The validation results confirm that, given a known PK model and dense, noise-free data, the absorption profile can be accurately estimated (Figure 4). The exception is the failure to capture the large initial peak in the long timescale data, which is only visible at the first data point (Figure 4, upper right). The method assumes that the data are noisy and considers that data point an outlier. Given that the function does not otherwise show any sharp peaks, this decision seems reasonable. In principle, this peak could be captured by even denser sampling, or by assuming a lower noise level. While such dense data are never obtained from real experiments, it is important to make sure that the method can handle this case before testing it on more realistic datasets.

When noise is added to the measurements, or more realistic sampling schedules are introduced, estimation accuracy decreases. Notably, in this problem, sparsity of sampling has a larger impact than noise for long timescales, while this is not the case for shorter timescales. This may partially be explained by the fact that at short timescales, the plasma concentration at any timepoint depends on the complete absorption rate profile up to that time. In that sense, the measurements provide information about the absorption rate between measurements. In contrast, over long timescales the plasma concentration essentially only provides information about the current absorption rate. Any inference on the absorption between measurements relies solely on the assumption that the function is smooth.

Input estimation of synthetic data could also be useful when designing future experiments. Using such simulations, it is possible to determine how the sampling affects the estimation accuracy, and one can then design a suitable sampling schedule accordingly.

The bioavailabilities obtained are substantially lower than previously reported values. However, the previous values refer to the bioavailability relative to a subcutaneous administration of an immediate-release formulation, making a direct comparison difficult to make. The differences may also partly be explained by uncertainty in the PK model. At least three PK models of exenatide have been published (Gao and Jusko, 2012; Chen et al., 2013; Li et al., 2015). These models are structurally similar but have substantially different parameter values. These parameters clearly influence estimated bioavailability—a drug with higher clearance requires higher absorption rates in order to maintain a specific plasma concentration. Also, in the Gao and Jusko model, the bioavailability of subcutaneous administration was fixed to 1, while the true bioavailability might be lower. In that case, the computed bioavailability would have to be rescaled to obtain the bioavailability relative to subcutaneous administration, in order to compare to previously reported values.

The estimation results demonstrate the extended and complex combined release/absorption kinetics of exenatide from PLG microspheres after subcutaneous injection. This is most clearly seen when the profiles are shown as absorption rate over time (Figures S2 and S3 in Supplementary Material) rather than as fraction absorbed over time (Figures 5 and 6). Immediately after injection, during the first couple of hours, a limited amount of exenatide is rapidly absorbed, corresponding to the release of freely available drug. It should be noted that the individual microspheres hydrate after injection and thereby tend to agglomerate to form an amalgam which will affect the release properties (DeYoung et al., 2011). The initial release is followed by an extended-release period of approximately 10 weeks where the polymer matrix of the PLG microspheres is slowly hydrolyzed to smaller fragments. The drug release rate is controlled by the diffusional transport of the drug through the polymer matrix and the erosion of the PLG depot system. The absorption rate over time profiles show two distinct peaks in the absorption rate, one at approximately 2 weeks and another at about 6–7 weeks after which the absorption rate declines until the PLG polymer is fully hydrolyzed and all the drug is released.

The choice of measurement noise model can have a large impact on the resulting estimates. An obvious default choice is to use a Gaussian noise model. However, as this model is log-quadratic, it will assign low probabilities to any candidate input function that disagrees significantly with even a single data point, making this model sensitive to outliers. Forcing the function to agree closely with every data point can drive the estimate of the regularization parameter to very low values. As a result, the method will assign high probabilities even to unrealistic, oscillatory functions, causing the reported uncertainty to be very high. Robust noise models using the Student’s *t*-distribution with a small number of degrees of freedom can decrease the sensitivity to outliers.

The methods that have been presented here are very general in that no mechanistic assumptions need to be made about the dissolution or absorption process. Still, some assumptions always have to be made when inferring a continuous-time function from a sparsely sampled and noisy dataset. In the input-estimation approach, these assumptions are encoded in the prior distribution of the stochastic process representing the input function. The prior chosen here, based on the norm of the second derivative, is only one of many possible choices. Previously, penalization of the first derivative (Verotta, 1996) and the use of entropic priors (Hattersley et al., 2008) have been suggested. The form of penalization of derivatives described here can also be viewed as a special case of the application of Gaussian processes, a rich class of probabilistic models for stochastic processes (Rasmussen and Williams, 2006). Depending on the application, other Gaussian processes may be more appropriate.

The number of MCMC samples required can be highly dependent on the data, even when the model remains unchanged. For the 7 mg dose, a much larger number of samples was required than for other doses, for both timescales. It can be noted that the 7 mg dataset contains possible outliers, suggesting that the presence of outliers could be an important factor in determining sampling efficiency. For most doses, a relatively modest number of MCMC samples was sufficient to obtain a high-quality estimate. This increases confidence in the applicability of these kinds of methods to ER-formulation research.

A strength of the methods presented here is that they are applicable even when the PK model is non-linear, but this obviously assumes that a model is available or can be constructed. In contrast, linear systems are completely characterized by their impulse response. The impulse response can be derived from a PK model, but it can also be estimated empirically from data. In that regard, non-linear systems require stronger assumptions to be made.

In Figure 1, all non-parametric modeling options can be viewed as special cases of input estimation. In that regard, any method that can handle the input-estimation case should be able to handle the other cases too. It may still be advantageous to use special-purpose methods for other cases. As an example, making a steady-state approximation removes the need to perform expensive numerical integration for each MCMC sample.

Input-estimation methods provide an attractive alternative to building a model of the release and absorption processes. Building such a model is non-trivial, and highly dependent on factors such as the type of delivery system and its geometry. In contrast, input-estimation methods strive to make minimal assumptions about these processes, requiring only that an intravenous PK model is available, which is independent of the formulation. In addition, these methods typically allow for more rapid analysis compared to building a mechanistic model. In this way, input estimation is a useful complement to model-based approaches.

In summary, this paper presents a framework for addressing input-estimation problems for drug-formulation development. It first gives an overview of what methods are available in various situations (Figure 1) and then puts emphasis on the most complicated case—non-parametric methods applied to dynamical systems with non-linear PK. The method presented in Trägårdh et al. (2016) is demonstrated to work robustly for a challenging ER test case with multiple peaks on various time scales, from hours to weeks, subject to the modifications required to cater for ER-formulation scenarios. The method provides estimates of the uncertainty, given the assumptions used in the statistical model. This has not previously been available. This helps increase confidence in the prediction of release and absorption rates. The predicted profiles make it possible to rank candidate formulations, predict the human PK, and establish an ivivc in order to minimize the need for *in vivo* studies. Additionally, we believe this approach has great potential from a practical perspective in supporting dose scheduling and regimens to yield optimal responses at the required times. Another possible application is to infer the release profile from data when a parametric model for the absorption of an immediate-release formulation is available, which could allow for a direct *in vitro* to *in vivo* comparison. This will be the topic of future work.

## Author Contributions

MT, MC, NE, JP, and PG initiated and planned the work. MT implemented and carried out the simulations, estimation, and analysis; drafted the manuscript, with input from the other authors. DJ performed the structural identifiability analysis. All the authors reviewed and approved the final manuscript.

## Conflict of Interest Statement

JP, DJ, and PG are employees of AstraZeneca R&D. MT is working together with Cardiovascular and Metabolic Diseases, Innovative Medicines and Early Development Biotech Unit, AstraZeneca but does not receive any funding from AstraZeneca.
