# Supplementary material for: Input Estimation for Extended-Release Formulations Exemplified with Exenatide
Source: Front Bioeng Biotechnol. 2017 Apr 19;5:24. doi: 10.3389/fbioe.2017.00024 (PMC5395652; doi:10.3389/fbioe.2017.00024)
Supplement: Supplementary file 1 [file presentation_1.pdf]

Supplementary material: Input Estimation for Extended Release Formulations Exemplified with Exenatide

Magnus Trägårdh, Michael J. Chappell, Johan E. Palm, Neil D. Evans, David L. I. Janzén, Peter Gennemark

*Correspondence:
Magnus Trägårdh
m.a.tragardh@warwick.ac.uk

1 STRUCTURAL IDENTIFIABILITY ANALYSIS

The structural identifiability of two PK models has been considered, Gao and Jusko (2012) and Li et al. (2015).

1.1 Structural identifiability analysis approaches

The structural identifiability analysis in this paper has been done using two approaches: The Exact Arithmetic Rank (EAR) and the Taylor series expansion approach.

The EAR approach was published in Karlsson et al. (2012) and for details of the method the reader is referred to that publication. The method is implemented as an automated Mathematica package and considers structural local identifiability. The user defines the model states, model parameters, initial conditions and input and output functions. These are used as arguments to the structural identifiability function defined within the Mathematica package which returns whether the model is locally identifiable or not. The method itself is based on local algebraic observability (Sedoglavic, 2002). Higher order derivatives of the output function(s) are generated resulting in a system of algebraic equations. The inverse function theorem is applied by checking whether the Jacobian matrix of the algebraic system is rank deficient or otherwise. If the Jacobian matrix of the algebraic system has full rank the model is structurally identifiable. All computations are carried out numerically, meaning that the model parameters and the initial conditions are numerically instantiated with randomly chosen integers. The input function is approximated with a truncated power series expansion with integer coefficients.

The Taylor series approach was first published in Pohjanpalo (1978) and for details of the method the reader is referred to that publication. This approach makes use of the fact that the coefficients in a power series expansion of a function uniquely determines the trajectory of that function. By computing the power series expansion of the output function and determine whether the model parameters can be uniquely determined from the coefficients in the power series expansion the structural identifiability of the model can be studied.

1.2 Gao and Jusko model

The structural identifiability analysis was done only of the PK model in Gao and Jusko (2012). In equation (1) in the paper, the variable R is used. This was interpreted as the total amount of free receptors at time t,

i.e., receptors available for drug binding at time t. Therefore, in the structural identifiability analysis the variable R was replaced with $(R_{tot} - RC(t))$.

The model structure of the PK model is given by

$$\begin{aligned}\frac{\mathrm{d}C(t)}{\mathrm{d}t} &= \frac{u(t)}{V_c} - (k_{el} + k_{pt}) \cdot C(t) + k_{tp} \cdot \frac{A_T(t)}{V_c} \\ &\quad - k_{on} \cdot (R_{tot} - RC(t)) \cdot C(t) + k_{off} \cdot RC(t) \qquad \text{(S1)}\end{aligned}$$

$$\frac{\mathrm{d}A_T(t)}{\mathrm{d}t} = k_{pt} \cdot C(t) \cdot V_c - k_{tp} \cdot A_T(t) \qquad \text{(S2)}$$

$$\frac{\mathrm{d}RC(t)}{\mathrm{d}t} = k_{on} \cdot (R_{tot} - RC(t)) \cdot C(t) - (k_{off} + k_{int}) \cdot RC(t) \qquad \text{(S3)}$$

where the unknown parameter vector is $\{V_c, k_{el}, k_{pt}, k_{tp}, k_{on}, k_{off}, k_{int}, R_{tot}\}$, $C(t)$ is the drug concentration in the central compartment, $A_T(t)$ is the drug amount in a peripheral compartment, $RC(t)$ is the concentration of the drug-receptor complex, and $u(t)$ is the unknown input.

1.2.1 Structural identifiability results

These were the results following from the structural identifiability analysis using the EAR approach.

- The PK model with bolus IV dose is structurally identifiable.
- The PK model with IV infusion is structurally identifiable.
- The PK model with SC administration is structurally unidentifiable with one degree of freedom. The unidentifiable parameters are F and V_c, the rest of the model parameters are identifiable.

Since all of the model parameters are shared for the IV case to the SC case, measures that can be taken to make the SC model identifiable include: *i)* fixing the parameter V_c to the estimated value from the IV case. *ii)* setting up a joint inference problem with both models.

In the paper the bioavailability parameter F was fixed to a value which solves the structural identifiability issue. However, this was not done as a direct result from a structural identifiability analysis.

1.2.2 Identifiability of the input function

In the analysis of whether the input function is identifiable all model parameters were assumed to be known.

To analyze whether the input function in the PK model in Gao and Jusko (2012) is identifiable or not the Taylor series expansion approach was used (Pohjanpalo, 1978). A Taylor series expansion of a function $y(t)$ around $t = 0$ is given by

$$y(t) = y(0) + y^{(k)}(0)\frac{t^k}{k!} + \ldots \qquad \text{(S4)}$$

where $k = 1, 2, \ldots$ All coefficients in the Taylor series determines uniquely the function $y(t)$. By computing the Taylor series expansion around $t = 0$ of the output function

$$y(t) = C(t) \qquad \text{(S5)}$$

we obtain the following coefficients:

$$
\begin{aligned}
y(0) &= 0 && \text{(S6)}\\
\dot{y}(0) &= u(0) && \text{(S7)}\\
\ddot{y}(0) &= u(0)\left(-\left(k_{\text{el}}+k_{\text{pt}}\right)\right) - u(0)k_{\text{on}}R_{\text{tot}} + \dot{u}(0) && \text{(S8)}\\
y^{(3)}(0) &= -\left(k_{\text{el}}+k_{\text{pt}}\right)\left(u(0)\left(-\left(k_{\text{el}}+k_{\text{pt}}\right)\right) - u(0)k_{\text{on}}R_{\text{tot}} + \dot{u}(0)\right)\\
&\quad - k_{\text{on}}R_{\text{tot}}\left(u(0)\left(-\left(k_{\text{el}}+k_{\text{pt}}\right)\right) - u(0)k_{\text{on}}R_{\text{tot}} + \dot{u}(0)\right) +\\
&\quad u(0)k_{\text{off}}k_{\text{on}}R_{\text{tot}} + u(0)k_{\text{pt}}k_{\text{tp}} + \ddot{u}(0) && \text{(S9)}\\
y^{4}(0) &= k_{\text{off}}k_{\text{on}}R_{\text{tot}}\left(-u(0)\left(k_{\text{el}}+k_{\text{pt}}\right) + u(0)\left(k_{\text{int}}-k_{\text{off}}\right) - u(0)k_{\text{on}}R_{\text{tot}} + \dot{u}(0)\right)\\
&\quad - \left(k_{\text{el}}+k_{\text{pt}}\right)\left(\left(k_{\text{el}}+k_{\text{pt}}\right)\left(u(0)k_{\text{el}} + u(0)k_{\text{on}}R_{\text{tot}} + u(0)k_{\text{pt}} - \dot{u}(0)\right) +\right.\\
&\quad k_{\text{on}}R_{\text{tot}}\left(u(0)k_{\text{el}} + u(0)k_{\text{on}}R_{\text{tot}} + u(0)k_{\text{pt}} - \dot{u}(0)\right) + u(0)k_{\text{off}}k_{\text{on}}R_{\text{tot}}+\\
&\quad \left.u(0)k_{\text{pt}}k_{\text{tp}} + \ddot{u}(0)\right) - k_{\text{on}}R_{\text{tot}}(\left(k_{\text{el}}+k_{\text{pt}}\right)\left(u(0)k_{\text{el}} + u(0)k_{\text{on}}R_{\text{tot}}\right.\\
&\quad \left.+ u(0)k_{\text{pt}} - \dot{u}(0)\right) + k_{\text{on}}R_{\text{tot}}(u(0)k_{\text{el}} + u(0)k_{\text{on}}R_{\text{tot}}\\
&\quad + u(0)k_{\text{pt}} - \dot{u}(0)) + u(0)k_{\text{off}}k_{\text{on}}R_{\text{tot}} + u(0)k_{\text{pt}}k_{\text{tp}} + \ddot{u}(0))-\\
&\quad k_{\text{pt}}k_{\text{tp}}\left(u(0)k_{\text{el}} + u(0)k_{\text{on}}R_{\text{tot}} + u(0)k_{\text{pt}} + u(0)k_{\text{tp}} - \dot{u}(0)\right) +\\
&\quad 3u(0)^2k_{\text{on}}^2R_{\text{tot}} + u^{(3)}(0) && \text{(S10)}\\
&\ \ \vdots
\end{aligned}
$$

The unknowns in the equations above are $\{u(0), \dot{u}(0), \ddot{u}(0), u^{(3)}(0), \dots\}$ since the model parameters are assumed to be known. Because of the structure of this particular model the $u^{(n-1)}(0)$ term will always enter linearly in the expression (see last term of each equation) for $y^{(n)}(0)$. As can be seen above, this results in a triangular structure of the higher order derivatives and we can therefore conclude that $\{u(0), \dot{u}(0), \ddot{u}(0), u^{(3)}(0), \dots\}$ can be determined from the output function.

The Taylor series expansion of the input signal $u(t)$ around $t = 0$ is

$$u(t) = u(0) + u^{(k)}(0)\frac{t^k}{k!} + \dots \tag{S11}$$

where $k = 1, 2, \dots$ Since we have shown that all of the coefficients in the Taylor series expansion of $u(t)$ can be determined we can conclude that the input function $u(t)$ is identifiable.

1.3 Li et al. model

The analyzed PK model in Li et al. (2015) has the following model structure

$$\dot{a}_1(t) = -k_{tr}a_1(t) \tag{S12}$$

$$\dot{a}_2(t) = -k_{tr}(a_1(t) - a_2(t)) \tag{S13}$$

$$\dot{a}_3(t) = -k_{tr}(a_2(t) - a_3(t)) \tag{S14}$$

$$\dot{a}_4(t) = -k_{tr}(a_3(t) - a_4(t)) \tag{S15}$$

$$\dot{a}_5(t) = -k_{tr}(a_4(t) - a_5(t)) \tag{S16}$$

$$\dot{a}_6(t) = k_a a_5(t) - \frac{Q}{V_1}a_6(t) - \frac{CL}{V_1}a_6(t) + \frac{Q}{V_2}a_7(t) - k_{on}a_8(t)a_6(t) + k_{off}a_9(t)V_1 \tag{S17}$$

$$\dot{a}_7(t) = \frac{Q}{V_1}a_6(t) - \frac{Q}{V_2}a_7(t) \tag{S18}$$

$$\dot{a}_8(t) = k_{syn} - k_{deg}a_8(t) - k_{on}a_8(t)\frac{a_6(t)}{V_1} + k_{off}a_9(t) \tag{S19}$$

$$\dot{a}_9(t) = k_{on}a_8(t)\frac{a_6(t)}{V_1} - k_{off}a_9(t) - k_{int}a_9(t) \tag{S20}$$

$$y(t) = \frac{a_6(t)}{V_1} \tag{S21}$$

with the unknown parameter vector $\{k_{tr}, k_a, k_{on}, k_{off}, k_{syn}, k_{deg}, k_{int}, Q, CL, V_1, V_2\}$. The structural identifiability analysis using the EAR approach showed that the PK model in Li et al. (2015) was structurally identifiable.

2 SYNTHETIC INPUT FUNCTION

The chosen synthetic input function $u(t)$ is a sum of three functions:

$$u(t) = u_1(t) + u_2(t) + u_3(t). \tag{S22}$$

where $u_1(t)$ and $u_2(t)$ are given by an Erlang distribution:

$$u_i(t) = a_i \frac{k_{tr_i}^{n_i} \cdot t^{n_i - 1} \cdot e^{-k_{tr_i}t}}{(n_i - 1)!} \quad i = \{1, 2\} \tag{S23}$$

and $u_3(t)$ is given by a biexponential function:

$$u_3(t) = r_1 e^{-k_1 t} + r_2 e^{-k_2 t}. \tag{S24}$$

The functions $u_1(t)$ and $u_2(t)$ can each be interpreted as the output of a chain of n_i compartments, connected with a rate constant of k_{tr_i}, where the initial value of the first compartment is a_i and all other compartments are initialized to 0. This rewriting is known as the “Linear Chain Trick” (Jacquez, 1996; Smith, 2010). Similarly, the initial biexponential $u_3(t)$ can be interpreted as the output from an absorption

compartment combined with a peripheral compartment. The complete input function can be generated by the model in Fig. S1. The parameters k_a, k_{12} and k_{21} in the figure are related to r_1, r_2, k_1 and k_2 via:

$$q = \sqrt{(k_{12} + k_{21} + k_a)^2 - 4k_{21}k_a} \tag{S25}$$

$$r_1 = \frac{k_a a_0}{2q}(q + k_{12} - k_{21} + k_a) \tag{S26}$$

$$r_2 = \frac{k_a a_0}{2q}(q - k_{12} + k_{21} - k_a) \tag{S27}$$

$$k_1 = \frac{1}{2}(q + k_{12} + k_{21} + k_a) \tag{S28}$$

$$k_2 = \frac{1}{2}(-q + k_{12} + k_{21} + k_a) \tag{S29}$$

where a_0 is the initial amount in compartment 1, and q is an intermediate variable to simplify the expressions. This result can be obtained by analytically solving the system of differential equations corresponding to compartments 1 and 2, and matching the coefficients. It might be more biologically plausible to connect $u_1(t)$ and $u_2(t)$ to the absorption compartment rather than directly to the central compartment. However, this would make little difference, since the parameter k_a is an order of magnitude larger than k_{tr_1} and k_{tr_2}, while making the algebraic expressions more complicated.

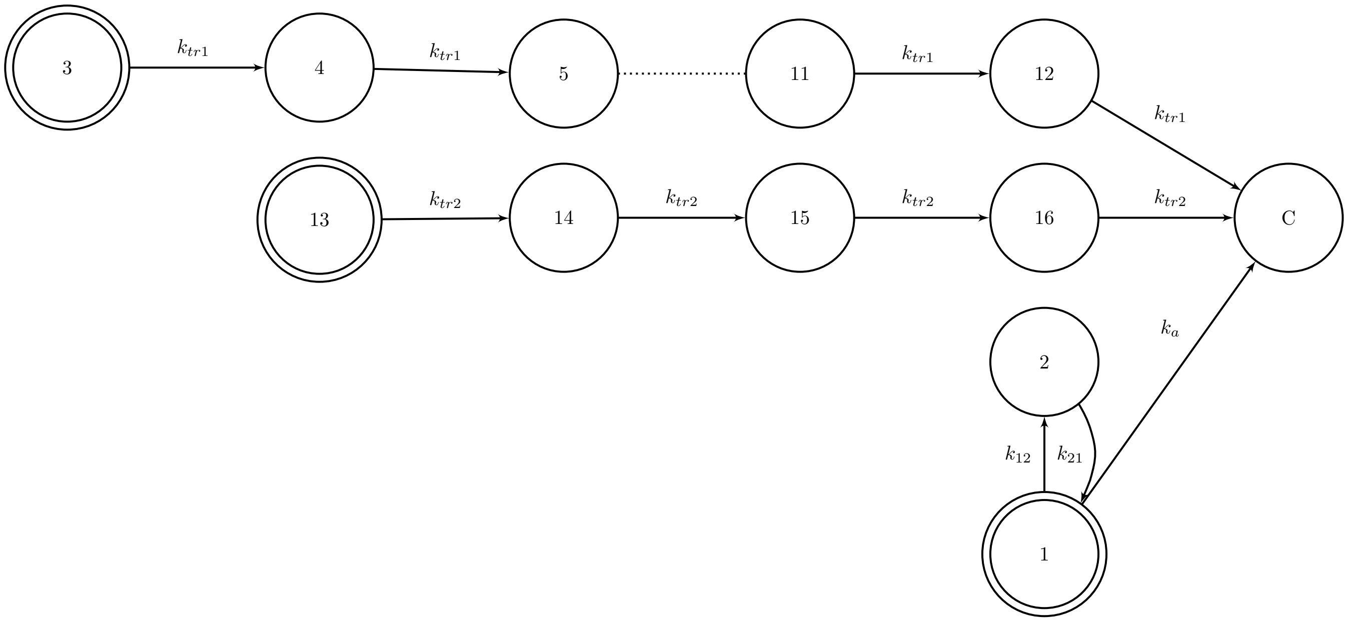

Figure S1. Compartmental model interpretation of the functions generating the test data that were used for assessing the performance of the estimation method. Compartments 1–2 generate the fast biexponential function accounting for the initial peak. The two chains 3–12 and 13–16 each produce an Erlang distribution-shaped function accounting for the peaks seen at longer timescales. Double circles indicate compartments with non-zero initial conditions. C is the central compartment. Note that compartments 6–10 are not shown.

3 ABSORPTION RATE PLOTS

The paper shows the estimation results for the real exenatide data expressed as the absorbed fraction of the drug as a function of time, as this is how such results are typically presented in the context of ER-formulation development. This appendix shows the same estimation results expressed as the absorption rate as a function of time, which can be useful for identifying the phases of drug absorption.

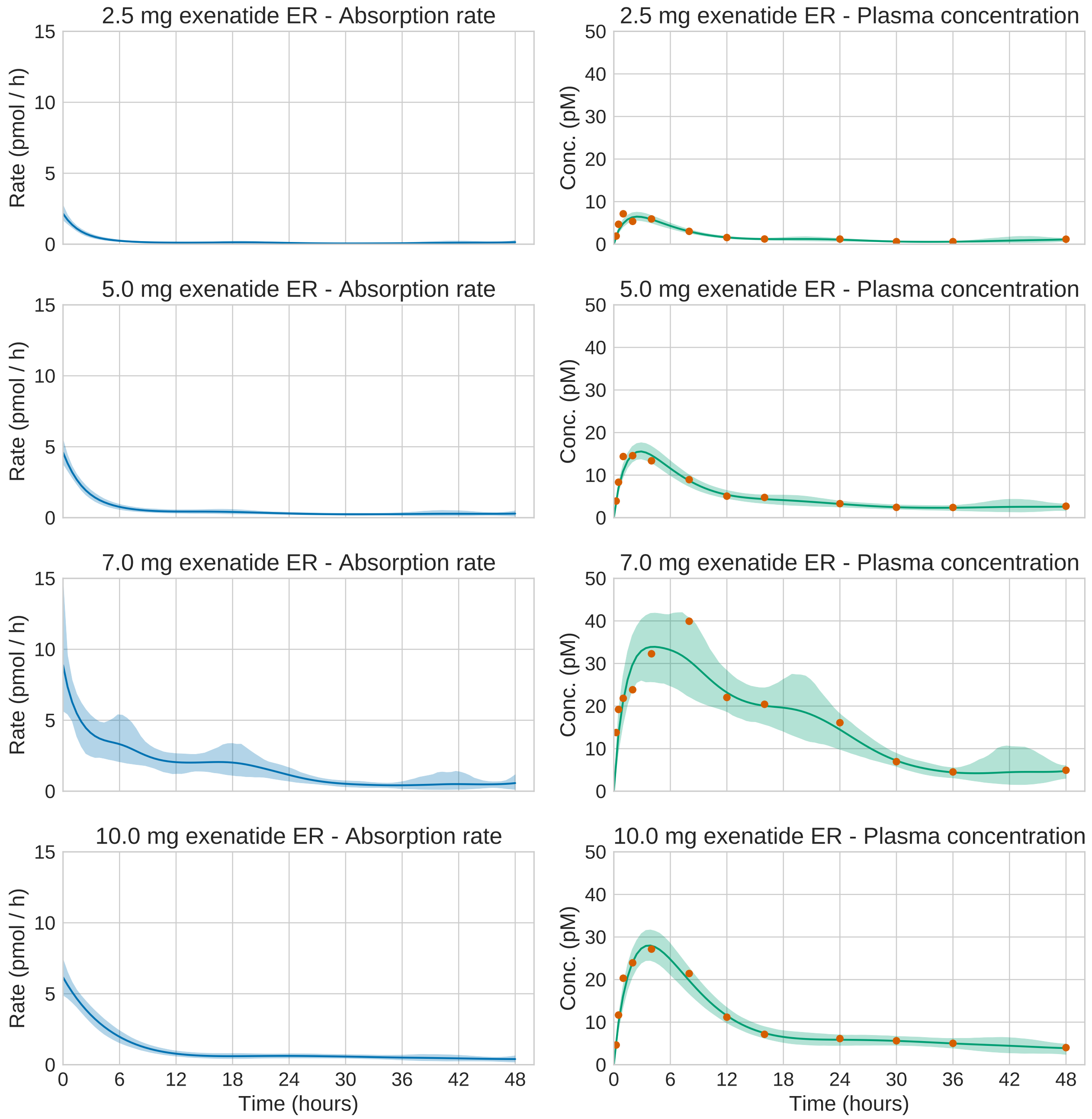

Figure S2. Estimation results including 95% credible intervals for the initial 48 hours of the real exenatide data, shown as absorption rate. It can be seen that the variations in the data for the 7.0 mg dose causes a larger uncertainty region.

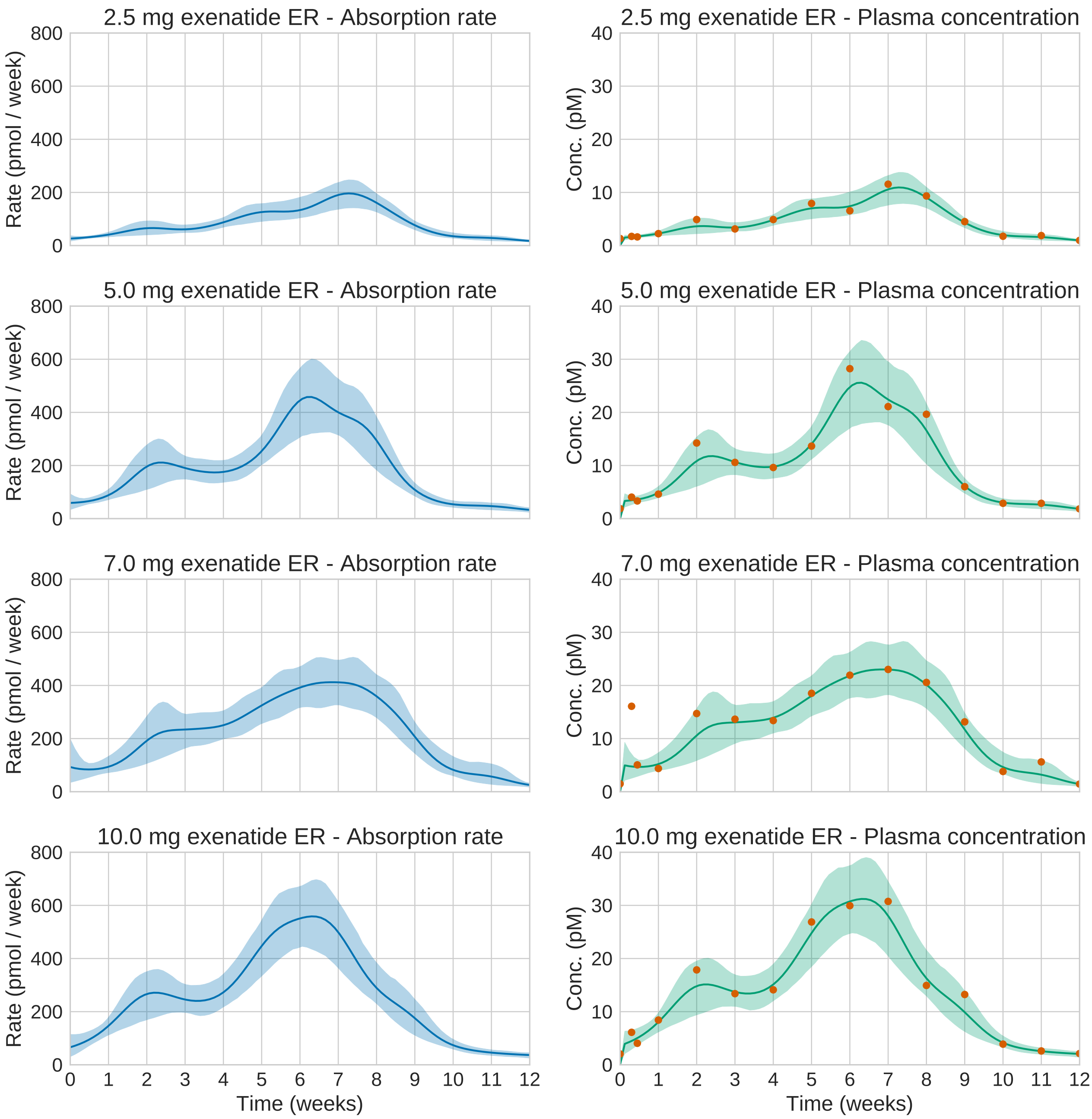

Figure S3. Estimation results including 95% credible intervals for the whole 12-week period of the real exenatide data, shown as absorption rate.

REFERENCES

Gao, W. and Jusko, W. J. (2012). Target-mediated pharmacokinetic and pharmacodynamic model of exendin-4 in rats, monkeys, and humans. *Drug Metabolism and Disposition* 40, 990–997

Jacquez, J. A. (1996). *Compartmental Analysis in Biology and Medicine* (Ann Arbor), third edn.

Karlsson, J., Anguelova, M., and Jirstrand, M. (2012). An efficient methods for structural identifiability analysis of large dynamic systems. *16th IFAC Symposium on system identification* , 941–946

Li, H., Xu, J., and Fan, X. (2015). Target-mediated pharmacokinetic/pharmacodynamic model based meta-analysis and dosing regimen optimization of a long-acting release formulation of exenatide in

patients with type 2 diabetes mellitus. *Journal of Pharmacological Sciences* 127, 170–180

Pohjanpalo, H. (1978). System identifiability based on the power series expansion on the solution. *Mathematical biosciences* 41, 21–33

Sedoglavic, A. (2002). A probabilistic algorithm to test local structural identifiability in polynomial time. *Journal of Symbolic computation* 33, 735–755

Smith, H. (2010). *An introduction to delay differential equations with applications to the life sciences*, vol. 57 (Springer Science & Business Media)
